# Scale Thickness Predicts Skin Puncture-Force Resistance in Three Pleuronectiform Fishes

**DOI:** 10.1093/iob/obz005

**Published:** 2019-04-08

**Authors:** M R Minicozzi, J Perez, D S Kimball, A C Gibb

**Affiliations:** Department of Biological Sciences, Northern Arizona University, Flagstaff, AZ 86011, USA

## Abstract

In fishes, the skin and scales provide a physical barrier to the external environment and must withstand direct physical insult from biotic and abiotic features of the habitat. Flatfishes likely rely heavily on their scales for physical defense because they rest directly on the substrate. Using a flatfish model, we asked: what are the effects of scale type and scale morphology on puncture force resistance? We also asked: are there morphological and functional differences between the eyed and blind sides in flatfishes and do the morphological and functional properties of scales vary with organism size? Using a large size range of three species of Pleuronectid flatfish (*Isopsetta isolepis*, *n* = 10; *Parophrys vetulus*, *n* = 10; and *Platichthys stellatus*, *n* = 12), we measured the force required to puncture the integument using a sample of skin+scales taken from the eyed and blind side of each individual. We also measured the diameter, area, and thickness of the scales of each individual. Scaling relationships (body length vs. variable of interest) were derived for each species and compared with *a priori* expectations of geometric similarity. We found no relationship between scale type and puncture resistance and no differences in morphological parameters or puncture resistance between the eyed and blind side within a given species. These flatfish species do vary in their ability to withstand puncture forces; however, once scale thickness is taken into account, species differences disappear. Thus, the ability of a flatfish to withstand mechanical insult from puncture-forces varies depending on the thickness of the scale.

## Synopsis

سمك القشر يتنبأ قوه مقاومه الثقب في الجلد في ثلاثة أسماك Pleuronectiformes في الأسماك ، يوفر الجلد والقشر حاجزًا ماديًا للبيئة الخارجية ويجب أن يتحمل الإهانة البدنية المباشرة من السمات الحيوية وغير الحيوية للموئل. يعتمد السمك المسطح بشكل كبير على القشر للدفاع عن النفس، لانهم يستقرون مباشرة على الطبقة التحتية. باستخدام نموذج السك المسطح سألنا: ما هي آثار نوع القشر و مورفولوجية القشر على مقاومة قوة ثقب؟ كما أننا سألنا: هل توجد فروق مورفولوجية ووظيفية بين جوانب العينين والجوانب العمياء في الأسماك المفلطحة وهل تختلف الخصائص المورفولوجية والوظيفية للقشر مع حجم الكائن الحي؟ باستخدام مجموعة كبيرة الحجم من ثلاثة أنواع من الأسماك المفلطحة ( *Isopsetta isolepis, n * = 10، *Parophrys vetulus *،, *n * = 10 and *Platichthys stellatus *،, *n * = 12) قمنا بقياس القوة المطلوبة لثقب التضمين باستخدام عينة من القشر و الجلد مأخوذة من الجانب العينين والمعمى لكل فرد. كما قمنا بقياس القطر والمساحة وسماكة القشر لكل فرد. وقد استمدت العلاقات التدريجية (طول الجسم مقابل متغير الاهتمام) لكل نوع وقورنت بالتوقعات المسبقة للتشابه الهندسي. لم نعثر على أي علاقة بين نوع القشر ومقاومة البزل ولا توجد فروق في المعلمات المورفولوجية أو مقاومة الثقب بين العينين والجانب العمياء داخل نوع معين. هذه الأنواع من الأسماك المسطحه تتفاوت في قدرتها على تحمل قوى الثقوب. ومع ذلك ، بمجرد أخذ سمك القشر بعين الاعتبار، تختفي اختلافات الأنواع. وبالتالي، فإن قدرة السمك المفلطح على تحمل الإهانة الميكانيكية من قوى الثقب تختلفباختلاف سمك القشر. Translation by A.A. Elbassiouny ( ahmed.elbassiouny@utoronto.ca )

El Grosor de la escala Predice la Resistencia a la Punción de la Piel en Tres Peces Pleuronectiformes (Scale Thickness Predicts Skin Puncture-Force Resistance in Three Pleuronectiform Fishes) En los peces, la piel y las escamas proporcionan una barrera física al ambiente externo y deben resistir la injuria física directa de las características bióticas y abióticas del hábitat. Los peces planos probablemente dependen mucho de sus escamas para la defensa física porque descansan directamente sobre el sustrato. Usando un modelo de peces planos, preguntamos: ¿cuáles son los efectos del tipo y la morfología de la escama en la resistencia a la fuerza de punción? También preguntamos: ¿hay diferencias morfológicas y funcionales entre los lados ciego y ocular en los peces planos y si las propiedades morfológicas y funcionales de las escamas varían con el tamaño del organismo? Usando un amplio rango de tamaño de tres especies de peces planos Pleuronectidos (*Isopsetta isolepis*, *n* = 10; *Parophrys vetulus*, *n* = 10; y *Platichthys stellatus*, *n* = 12), medimos la fuerza requerida para perforar el integumento utilizando una muestra de escamas de la piel tomada del lado ocular y ciego de cada individuo. También medimos el diámetro, el área y el grosor de las escamas de cada individuo. Se derivaron relaciones escalares (longitud corporal vs. variable de interés) para cada especie y se compararon con las expectativas *a priori* de similitud geométrica. No encontramos ninguna relación entre el tipo de escama y la resistencia a la punción, y no hay diferencias en los parámetros morfológicos o la resistencia a la punción entre el lado ciego y ocular dentro de una especie dada. Estas especies de peces planos varían en su capacidad para soportar las fuerzas de punción; sin embargo, una vez que se toma en cuenta el grosor de la escama, las diferencias entre especies desaparecen. Por lo tanto, la capacidad de un pez plano para soportar la injuria mecánica de las fuerzas de punción varía según el grosor de la escama. Translated to Spanish by C.A. Alfonso (calfonsoc@vt.edu)

Die Schuppendicke sagt die Durchstoßfestigkeit der Haut bei drei Pleuronectiformen Fischen Voraus (Scale Thickness Predicts Skin Puncture-Force Resistance in Three Pleuronectiform Fishes) Bei Fischen stellen die Haut und die Schuppen eine physische Barriere für die äußere Umgebung dar und müssen einer direkten physischen Belastung durch biotische und abiotische Faktoren des Lebensraums standhalten. Plattfische sind für ihre physische Verteidigung wahrscheinlich stark auf ihre Schuppen angewiesen, da sie direkt auf dem Substrat verweilen. Anhand eines Plattfischmodells stellten wir die Frage: Was sind die Auswirkungen des Schuppentyps und der Schuppenmorphologie auf die Durchstoßfestigkeit? Wir haben auch gefragt: Gibt es morphologische und funktionelle Unterschiede zwischen den Augen- und Blindseiten der Plattfische und variieren die morphologischen und funktionellen Eigenschaften der Schuppen mit der Größe des Organismus? Unter Berücksichtigung eines breiten Größenspektrums von drei Arten von Pleuronectiden (*Isopsetta isolepis*, *n* = 10; *Parophrys vetulus*, *n* = 10; und *Platichthys stellatus*, *n* = 12) wurde die Kraft gemessen, die erforderlich ist, um das Integument zu punktieren, an einer Probe von Haut und Schuppen, jeweils von der Augen- und Blindseite. Wir haben auch den Durchmesser, die Fläche und die Dicke der Schuppen jedes Individuums gemessen. Skalierungszusammenhänge (Körperlänge vs. Variable von Interesse) wurden für jede Spezies abgeleitet und mit den *a priori* Erwartungen der geometrischen Ähnlichkeit verglichen. Wir fanden keinen Zusammenhang zwischen Schuppenart und Durchstoßfestigkeit und keinen Unterschied in den morphologischen Parametern oder der Durchstoßfestigkeit zwischen der Augen- und Blindseite innerhalb einer gegebenen Spezies. Diese Plattfischarten unterscheiden sich allerdings in ihrer Fähigkeit, Durchstichkräften zu widerstehen; sobald jedoch die Schuppendicke berücksichtigt wird, verschwinden die Artenunterschiede. Daher variiert die Fähigkeit eines Plattfisches, einer mechanischen Einwirkung durch Durchstichkräfte standzuhalten, abhängig von der Dicke der Schuppe. Translated to German by F. Klimm (frederike.klimm@neptun.uni-freiburg.de)

Espessura da Escama Prevê a Resistência à Força de Punção Cutânea em Três Peixes Pleuronectiformes (Scale Thickness Predicts Skin Puncture-Force Resistance in Three Pleuronectiform Fishes) A pele e as escamas dos peixes fornecem uma barreira física ao ambiente externo, devendo aguentar injúrias provenientes dos aspectos bióticos e abióticos do seu habitat. Os linguados provavelmente dependem muito de suas escamas para defesa porque eles estão em contato direto com o substrato. Usando um modelo de linguado, perguntamos quais os efeitos do tipo de escama e da morfologia da mesma na resistência à punção. Nós também perguntamos se existem diferenças morfológicas e funcionais entre os lados dos olhos e os lados cegos em linguados e se as propriedades morfológicas e funcionais das escamas variam com o tamanho do organismo. Usando uma extensa faixa de tamanho de três espécies de linguados pleuronectídeos (*Isopsetta isolepis*, *n* = 10; *Parophrys vetulus*, *n* = 10; e *Platichthys stellatus*, *n* = 12), medimos a força necessária para perfurar o tegumento em uma amostra de pele+escamas tirada de ambos os lados de cada indivíduo. Também medimos o diâmetro, área e espessura das escamas deles. As relações de escalonamento (comprimento do corpo vs. variável de interesse) foram derivadas para cada espécie e comparadas às expectativas de similaridade geométrica determinadas *a priori*. Não encontramos relação entre o tipo de escama e a resistência à perfuração e não houve diferenças nos parâmetros morfológicos ou na resistência à perfuração entre o lado dos olhos e o lado cego em uma dada espécie. Essas espécies de linguado variam em sua capacidade de aguentar forças de punção; no entanto, uma vez que a espessura da escama é levada em conta, as diferenças entre as espécies desaparecem. Assim, a capacidade de um linguado de suportar injúrias mecânicas causadas por perfuração varia dependendo da espessura da escama. Translated to Portuguese by G. Sobral (gabisobral@gmail.com)

## Introduction

In the ray-finned, or actinopterygian, fishes, the skin and scales form the integument, which mediates the interaction between the animal and the external environment. Because ray-finned fish species live in a wide variety of environments—pelagic (open-ocean), benthic (bottom-dwelling), and in some cases even terrestrial (on land) habitats—scales have been modified to serve distinct purposes. In some fishes, such as *Lepidopsetta polyxystra* (northern rock sole), scales may enhance crypsis (Ryer et al. 2004). For many pelagic species, scales reduce the friction drag experienced as a fish swims through the water (Fish 1998). In benthic species, scales may serve to increase friction between the animal and the substrate (Spinner et al. 2016), a feature that could improve a fish’s ability to entrap substrate particles on top of the body, thus disguising its presence. Beyond the functions listed above, scales and skin must resist mechanical forces that could puncture the fish’s integument.

Among fishes, flatfishes are unusual because they are bilaterally asymmetrical and spend their lives resting on the bottom, with one side of the body in direct contact with the substrate. Flatfishes hatch as symmetrical larvae, but one eye migrates over the top of the head to the opposite side of the body during metamorphosis. As a result, in flatfish adults, one side is “eyed” (two eyes) and the other side is “blind” (eyeless) and a flatfish typically rests on the bottom with the blind side in direct contact with the substrate. Adult flatfishes are asymmetrical in the jaws and paired fins, and flatfishes are also typically unpigmented on the blind side of the body. Most flatfish species have either embedded (under the epidermis) cycloid scales or overlapping ctenoid scales, but occasionally they have protruding, tuberculate scales, as seen in the genus *Platichthys* (Roberts 1993).

Flatfishes are a useful model system in which to study functional aspects of scales because the environment can potentially impose different selective forces on the eyed vs. the blind side of the body. When burying in the substrate, flatfish cyclically oscillate the entire body in close proximity to the granular medium that comprises the substrate. Thus, as a normal part of a flatfish’s life history, the ventrally-oriented blind-side is repeatedly forced against, rough, potentially damaging particles. In addition, the blind side of a flatfish is vulnerable to a variety of parasites or predators that live in the benthos and attack the fish from below, while the eyed side of the fish faces a different set of challenges, including free-living aquatic predators and a distinct cadre of parasites that may attack fish from the water column. Indeed, the scales of some flatfish species are markedly different on one side of the body versus the other. For example, *Citharichthys sordidus*, the Pacific sanddab, has ctenoid scales on the eyed side, which could serve to reduce friction drag with the water flowing over the fish, but possesses cycloid scales on the blind side where the body is in direct contact with the substrate (Batts 1964). In contrast, although *Pleuronectes platessa*, the European plaice, possesses cycloid scales on both the eyed and blind sides of the body, the eyed-side scales have been demonstrated to generate greater frictional forces when interacting with the sediment, relative to blind-side scales (Spinner et al. 2016). Thus, the scales on the eyed vs. the blind side are *functionally* different from one another in *P. platessa*, even though they are of the same type.

The ability of the integument to resist puncture forces is a function of the material properties of the cells and connective tissues that form the skin and scales (Ikoma et al. 2003), in combination with properties of the structure that is attempting to penetrate the integument. For example, shark teeth (Whitenack and Motta 2010), insect proboscises (Oka et al. 2002), or cactus spines (Crofts and Anderson 2018) all have been demonstrated to pose distinct mechanical challenges to biological materials. Indeed, material scientists have documented the properties of offensive and defensive structures and used this information when designing microneedles (Oka et al. 2002), flexible armors (Zhu et al. 2013), and other human-serving devices (Chen et al. 2012).

Puncture resistance of defensive structures such as fish scales is likely to be influenced by the three-dimensional shape of the scale: length, width, thickness, and overall topography as well as intrinsic material properties. Of the shape parameters, scale depth is perhaps likely to be the most important, as puncture resistance has been hypothesized to be strongly influenced by scale thickness (Ikoma et al. 2003; Whitenack and Motta 2010; Bergman et al. 2017). Assuming that scale thickness varies in proportion to animal size (i.e., assuming scale thickness is directly proportional to total fish length), then resistance to puncture forces is expected to vary in direct proportion with body size. Under this assumption, we would predict that, as a fish doubles in length, the ability of the animal’s integument to withstand puncture forces will also approximately double. However, we note that it is possible that scale thickness varies among species, with some species having markedly thicker scales, or that the relationship between fish size and scale thickness is not consistent (varies) among species.

In this study, we quantified the relationship between body size, scale morphology, and puncture-force resistance in three species of pleuronectid (family Pleuronectidae) flatfishes from the Northeastern Pacific Ocean. To investigate how scale type may influence morphological (e.g., thickness) and functional (e.g., puncture-resistance) characteristics, we chose three species with different scale types. *Parophrys vetulus* (English sole) has cycloid scales, *Isopsetta isolepis* (butter sole) has ctenoid scales, and *Platichthys stellatus* (starry flounder) has tuberculate scales (Roberts 1993; Batts 1964). Because *Platichthys* have prominent, physically pointed, tuberculate scales, which suggests that they are, in some sense, “armored,” we predicted that *Platichthys* would have the greatest puncture-resistance of the three species considered here. However, we also expected that puncture resistance would be heavily influenced by scale thickness, because the thickness of a scale represents the dimension of a scale that contains the material that resists compressive forces applied to the body.

By quantifying body size, scale dimensions, and puncture resistance in three flatfish species with different scale types, we sought to answer the following questions. (1) Do scales of a given flatfish species demonstrate the same morphological dimensions and functional properties on the eyed vs. blind sides? (2) Does the relationship between scale morphology/performance vary with body size in a consistent manner across species? (3) Do scales of different types (cycloid, ctenoid, tuberculate) appear to have different abilities to resist puncture forces? (4) Alternately, is the ability to resist puncture-forces best explained by simple morphological parameters—particularly scale thickness? To answer these questions, we examined a size range individuals representing three flatfish species to enable us to calculate intraspecific scaling (proportional) relationships for body size, scale dimensions, and performance parameters. A scaling-based approach enables us to determine if key characteristics of flatfish scales, such as thickness, remain consistent within a species individuals grow larger and if the relationship between size and morphology/function is the same in the three flatfish species considered here.

## Materials and methods

Individuals representing three species of flatfish (*I.isolepis*, *n* = 10 and *P.vetulus*, *n* = 10 and *P.stellatus*, *n* = 12) were collected from otter trawls and beach seins conducted in the Salish Sea (Puget Sound, WA, USA). Individuals were maintained in flow-through sea tables at Friday Harbor Laboratories on a 16/8 light dark cycle at 16 ± 2°C and fed Pandalid shrimp daily. Prior to the puncture-force trials, total length of the animal was taken, and then the animal was euthanized via an overdose of buffered Tricaine mesylate (MS-222).

After animals were euthanized, a sample of skin and embedded scales was removed from a region just dorsal and posterior to the pectoral fin (along the lateral line) on the blind and eyed side of each flatfish. After the skin sample was excised, most of the underlying musculature was removed. To provide a flesh-like base for the puncture-force trials, the section of excised skin + scales was stretched over a section of an apple, selected to mimic the softer tissues that underlie the integument (modulus of apple =2.0–3.3 [Grotte et al. 2002] and modulus of muscle =1.5–3.1 [Chen et al. 1996]), and pinned into place.

**Fig. 1:**
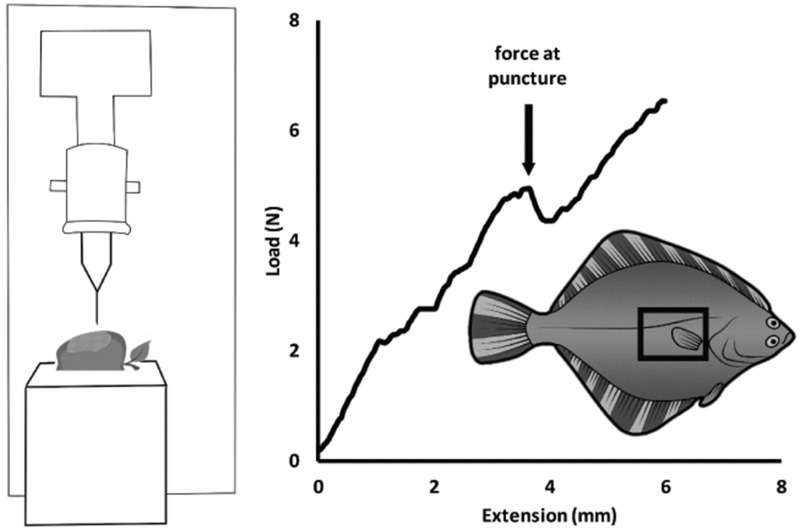
Experimental methodology. Skin+scales were removed from just behind the pectoral fin on both the eyed and blind sides of the body and then stretched over an apple, which was selected to mimic the material properties of muscle tissue. An insect pin attached to a MTS was lowered through the skin and into the apple. In these experiments, force consistently increased until the pin punctured the scales, causing an abrupt decline in force.

For the puncture trials, a #4 insect pin (tip diameter 40–50 µm) was attached to a 500 N load-cell of a Synergie 100 material testing system (MTS) and used to puncture the flatfish skin. The pin in the MTS was positioned approximately 2–5 mm above the scale and the pin was lowered to the sample at a speed of 100 mm min^−^^1^. An insect pin was chosen to provide a consistent mechanical insult that can be used as a proxy for many types of fine-scale injury that could potentially occur in the fish’s habitat, such as small teeth, crustacean appendages, or abrasion from sand or other coarse substrates. Each piece of skin was punctured two to five times and a new location on the sample (a new scale) was targeted during each puncture test; puncture resistance was sampled in multiple locations to determine the maximum puncture resistance for a given skin sample. Each skin sample was punctured with a new pin to reduce the effects of wear of the pin. Puncture force was determined as the maximum force value right before a sharp decrease in force (Fig. 1). All MTS data were analyzed on TestWorks 2003 and the largest force measured for a given piece of skin was used for statistical analyses.

**Fig. 2:**
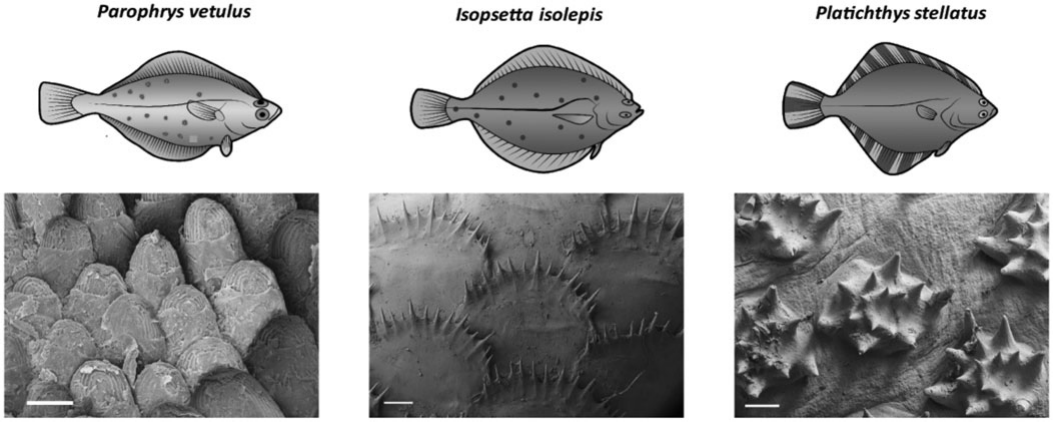
Each flatfish species possesses a different scale type. Scanning electron microscope images of the eyed side skin and scales of each flatfish species confirm that *P. vetulus* display embedded, cycloid scales, *Isopsetta isolepis* display projecting, ctenoid scales, and *P. stellatus* display spiny, “tuberculate” scales.

Scale morphology and anatomical dimensions (morphometrics) were analyzed from a subset of individuals used in the puncture force trials (*Parophrys*, *n* = 8; *Isopsetta*, *n* = 6; and *Platichthys*, *n* = 9). When the skin samples were obtained from small individuals, morphometrics and scanning electron microscope (SEM) images were obtained from the punctured skin following the material testing. In large individuals, a second skin sample was collected just posterior to the sections used in the puncture-force trials. SEM was used to visualize surface morphology, and images were collected of the skin section at 0° (directly overhead) and at 45° rotation (an oblique angle). For the tissues used in SEM, skin and scales were fixed in 70% ethanol for several days before being dehydrated in a graded ethanol series. The fish were then critical point dried in a Pelco CPD-2 critical point dryer and mounted on aluminum stubs using carbon tape. Samples were then sputter-coated with gold/palladium for 1 min in a Denton Vacuum Desk II sputter coater and viewed with a Zeiss Supra 40VP SEM at 5 kV. Skin and scale samples for morphometric analyses were preserved in 70% ethanol and photographed using a Zeiss stereomicroscope with an AxioVison camera and AxioVision Rel 48 software. Images were subsequently analyzed using NIH ImageJ. Because there is overlap in adjacent scales in some species, individual scales were removed from *Parophrys* and *Isopsetta* to enable measurements of the diameter and area of the scales (Supplementary Fig. S1). However, there was no overlap in the scales of *Platichthys* (Fig. 2), so the scales were measured *in situ*.

After measurements of diameter and area were taken, sections of skin samples were processed and embedded in paraffin wax using a Shandon Citadel 2000 tissue processor. Each skin sample was sliced at 20 microns, and scale thickness was measured from these sections (Supplementary Fig. S1). Because scales tend to be thickest at the focus (center), serial sections of the skin and scales were measured multiple times along the same scale to determine the thickest region. Thickness was calculated in this manner for three to five scales for each individual on each side of the body and the largest number was used in the scaling analysis.

The relationships between body size, scale morphometrics, and puncture-force resistance were calculated by taking the log (base 10) of the following variables: standard length, scale diameter (smallest axis of the scale), scale area (total surface area), scale thickness, and puncture force. The slope of the regression between length and the other variables was calculated as the scaling exponent (*y* =*ax*^b^). Note that all data given in figures are presented using untransformed variable values and expressed on log–log axes to allow intuitive visual interpretation of the data. However, all statistical analyses were conducted using log-transformed data.

Potential differences between the eyed and blind side of each species were evaluated using a paired samples *t*-test in IBM SPSS statistics ver. 24. Data were analyzed to evaluate the assumptions of normality (Shapiro–Wilk) and homogeneity of variance (Levene’s). All variables (log transformed) were normal except log-length for *Isopsetta* (*w* = 0.824, df = 12, *P* = 0.018) and scale log-thickness for *Isopsetta* (*w* = 0.716, df = 12, *P* < 0.001). Although these data are not normally distributed, the analyses performed here are not as sensitive to the normality assumption as other statistical tests (Box 1953; Lumley et al. 2002) and all variables (log transformed) displayed homogeneity of variance (all *P*-values > 0.14). We found that the morphological and performance variables for the eyed vs. blind sides were not statistically different for any of the three species (*P* > 0.05, Supplementary Table S1). Therefore, the eyed and blind side data were combined (pooled) for a given species in all subsequent scaling analyses.

To test the null hypothesis that there is no change in the proportional size of the scales as fish grow larger (the hypothesis of geometric similarity or isometry [Hill 1950]) the slopes for the log of each variable vs. log size were compared with the predictions of geometric similarity. Under the assumptions of geometric similarity, all linear measurements (length, width, thickness) should scale with body length with a slope of 1 (L^1^); all area measurements should scale with standard length with a slope of 2 (L^2^). Student’s *t*-tests were used to test the null hypothesis that there is no difference between the empirically determined scaling relationships and the scaling slope for each variable as predicted by the assumptions of geometric similarity.

Log-transformed variables were also analyzed using ANCOVA (in IBM SPSS Statistics 24) to assess the effects of species and size (using specimen length as a covariate) for each performance and morphological variable (puncture force, scale diameter, scale area, and scale thickness). The interaction between size and species was used to determine if the slopes were equivalent across all species, where a significant size-by-species interaction term indicated that the slopes varied across taxa. We also conducted ANCOVA using species as the fixed factor, thickness as a covariate, and puncture force as the dependent variable. This ANCOVA assessed the relationship between scale thickness and puncture force across species, where a significant interaction term would indicate that the relationship between scale thickness and puncture force varies among the three flatfish species considered here.

## Results

Light and electron microscopy confirmed that each species possesses a different scale type, but also revealed that scale type did *not* vary on the blind vs. eyed sides for any of the species considered here. *Parophrys* have small, embedded cycloid scales on the eyed and blind sides. *Isopsetta* have large projecting ctenoid scales on the eyed and blind sides. *Platichthys* have large tuberculate scales on both the eyed and blind sides, and these scales are characterized by numerous projecting ctenial spines (Fig. 2). In addition, no morphometric (dimensional) variable differed between eyed and blind sides (all *P*-values > 0.05, Supplementary Table S1). In addition, puncture force did not differ between the eyed and blind sides for any of these three species (Supplementary Table S1). Eyed and blind side data were combined (pooled) for a given species in all subsequent statistical analyses.

**Fig. 3:**
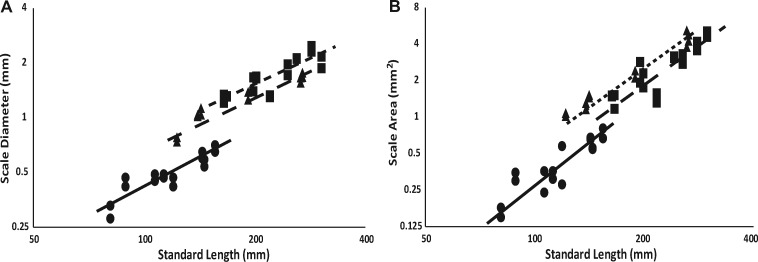
*Isopsetta* display different scaling relationships for scale diameter and area than *Parophrys* and *Platichthys*. Here, standard length is plotted against scale diameter (**A**) and scale surface area (**B**), with representative regression lines for each species indicated on the graphs. Axes are log-scaled to allow intuitive interpretation of scaling relationships, all statistics were performed on log-scaled data. *Parophrys* individuals are represented by circles and solid line (*n* = 16), *Platichthys* individuals are represented by squares and small dashed line (*n* = 18), and *Isopsetta* individuals are represented by triangles and large dashed line (*n* = 12).

For scale morphometrics, we tested the assumptions of geometric similarity. For scale diameter and area we tested the null hypothesis that these parameters would scale with body length to the first power (L^1^) and that scale area should scale with body length to the second power (L^2^). We found that *Parophrys* and *Platichthys* display isometric (linear) scaling (slope of 0.96 and 0.93, *t* =−0.8, df = 14, and *t* =−1.3, df = 16, respectively, *P* > 0.05) for scale diameter, while *Isopsetta* displays sublinear scaling (slope of 0.86, *t* =−3.5, df = 10, *P* = 0.006; Fig. 3A; Table 1). Scale area shows a similar trend, where *Parophrys* and *Platichthys* display isometric scaling (slopes of 1.96 and 1.90 *t* =−0.4, df = 14 and *t* =−1.0, df = 16, respectively, *P* > 0.05) while *Isopsetta* displays sublinear scaling (slope of 1.86, *t* =−3.3, df = 10, *P* = 0.008; Fig. 3B; Table 1).

**Fig. 4:**
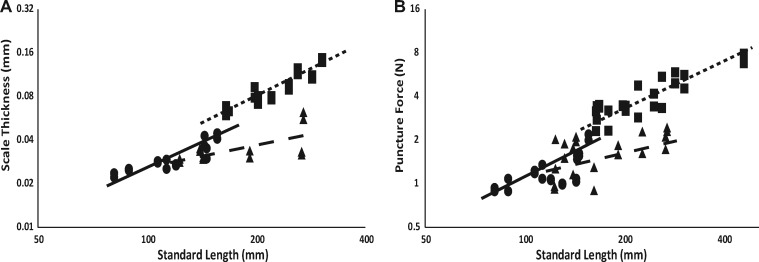
Scale thickness appears to be the best predictor of puncture resistance. Here, standard length is plotted against scale depth (**A**) and scale puncture force (**B**), with representative regression lines for each species indicated on the graphs. Axes are log-scaled to allow intuitive interpretation of scaling relationships, all statistics were performed on log-scaled data. *Parophrys* individuals are represented by circles and solid line (*n* = 16 for depth and *n* = 20 for puncture force), *Platichthys* individuals are represented by squares and small dashed line (*n* = 18 for depth and *n* = 24 for puncture force), and *Isopsetta* individuals are represented by triangles and large dashed line (*n* = 12 for depth and *n* = 20 for puncture force).

Scale thickness in the three species demonstrates a different pattern. *Parophrys* exhibit approximately linear (isometric) scaling (slope of 0.83, *t* =−0.4, df = 14, *P* = 0.73), *Platichthys* displays moderate supralinear scaling (a slope of 1.2, *t* = 4.3, df = 16, *P* < 0.001), and *Isopsetta* displays marked sublinear scaling (slope of 0.49, *t* =−11.6, df = 10, *P* < 0.001, Fig. 4A; Table 1). Our *a priori* hypothesis was puncture resistance would be largely dictated by scale thickness; thus, we would expect puncture resistance to show similar patterns to those of scale thickness. This hypothesis was supported: puncture resistance scales linearly with body size in *Parophrys* and *Platichthys* (slopes of 0.85 and 0.91, *t* =−1.8, df = 18 and *t* =−1.2, df = 22, respectively, *P* > 0.05), but shows marked sublinear scaling in *Isopsetta* (slope of 0.56, *t* =−3.7, df = 18, *P* = 0.001, Fig. 4B; Table 1).

**Table 1:** Empirically-derived scaling relationships for each flatfish species for three morphological variables and puncture resistance

	*Parophrys vetulus*			*Isopsetta isolepis*			*Platichthys stellatus*		
	Slope	*a*	*R* ^2^	Slope	*a*	*R* ^2^	Slope	*a*	*R* ^2^
Scale diameter	0.96	−1.69	0.8	0.86*	−1.05	0.91	0.93	−1.56	0.74
Scale area	1.96	−4.46	0.79	1.86*	−3.87	0.98	1.9	−4.08	0.77
Scale thickness	0.83	−2.3	0.83	0.49*	−1.86	0.5	1.2*	−1.96	0.86
Puncture resistance	0.85	−3.24	0.49	0.56*	−2.57	0.3	0.91	−3.89	0.75

Asterisk denotes statistical difference from predictions of isometric scaling. Scale diameter, scale thickness, and puncture-force resistance were compared with the hypothesized isometric slope of 1, while scale area was compared with the hypothesized isometric slope of 2.

ANCOVA indicated that the relationship (slope) between size and morphological and performance variables is consistent across species for scale diameter, scale area, and puncture force, but not for scale thickness (Table 2). A full ANCOVA considering species as the independent variable, body size as the covariate, and puncture force as the dependent variable (removing the non-significant interaction term) revealed a significant species effect (*F* = 46.496, df = 2, *P* < 0.001); this suggests that puncture forces are different when species are corrected for body size. However, the slopes for scale thickness were different for each species; thus, the relationship between body size and scale thickness was *not* consistent across taxa. Instead, *Parophrys* and *Platichthys* scale linearly as they grow larger while *Isopsetta* scales sublinearly as they grow larger. When ANCOVA is conducted using species as the independent variable, scale thickness as the covariate, and puncture force as the dependent variable (all assumptions were met for this analysis: homogeneity of regression *F* = 1.691, df = 2, *P* = 0.198) and the non-significant interaction term is removed, there was no effect of species. Thus, the force required to puncture a scale is actually the same across all three species if the data are corrected for scale thickness (*F* = 1.181, df = 2, *P* = 0.318).

**Table 2:** ANCOVA of morphological and performance variables considering species as the independent variable and length as a covariate

Dependent variable	*F*	*df*	*P*-value
Scale diameter	0.214	2	0.810
Scale area	0.057	2	0.950
Scale thickness	5.887	2	0.006*
Puncture force	1.381	2	0.261

*F*, df, and the *P*-value represent the results of the interaction term of length * species for each dependent variable. Asterisk denotes a significant interaction and a violation of the assumptions of ANCOVA.

## Discussion

Because previous studies of flatfish scales reported morphological (Batts 1964) and functional (Spinner et al. 2016) differences between the two sides of the body, we predicted there would be functional differences between the eyed and blind sides of the flatfish species considered here. However, we found no bilateral differences in any aspect of morphology for these three species: scales on the two sides of a fish were not different in scale type (cycloid vs. ctenoid vs. tuberculate) or in scale dimensions. Correspondingly, we found no difference for eyed and blind side scales for a key functional parameter: resistance to puncture force.

Based on previous work (Whitenack and Motta 2010; Bergman et al. 2017), we expected that puncture resistance would be directly proportional to scale thickness, where thicker scales require greater forces to puncture. Our results support this prediction, as the variation in scale thickness appears to explain the variation in puncture resistance in all three species. In fact, when scale thickness is considered a covariate, all species have similar ability to resist puncture forces. However, although thickness may largely determine puncture resistance, other factors not considered in this study could contribute to the ability of the fish’s integument to resist puncture. For example, the material properties, such as the degree of ossification or the cellular structure of lamellar bone, could be modified to change puncture resistance. In addition, although to a much lesser extent, factors such as collagen fiber orientation of the scales (Okuda et al. 2011) or thickness of the fish’s skin, could also affect the resistance to puncture forces.

One unexpected finding of this study was that *Isopsetta* scales grow proportionally “thinner” as the individuals of this species become larger. Fish scales can represent a large portion of a fish’s biomass; this is particularly true for flatfish because the laterally-compressed body shape yields increased surface area. It is possible that *Isopsetta* seek to reduce their metabolic expenditure in creating and maintaining scales in an effort to grow larger at a faster rate. However, differences in scale thickness could also represent different anti-predator strategies—where *Isopsetta* relies more on crypsis, as opposed to physical armor.

Surprisingly, scale type appears to have little effect on resistance to puncture force. Although the pointy tuberculate scales of *Platichthys* give it the appearance of being “armored,” the cycloid scales of *Parophrys* that are the same thickness as *Platichthys* scales have a similar level of puncture-force resistance. The tuberculate scales of *Platichthys* were originally of interest in this study because of their unusual morphology. Yet, this unusual morphology did not appear to provide any increase in puncture force resistance. However, because each species possessed a different scale type, we cannot distinguish species differences from scale-type differences. In addition, we cannot rule out effects of different failure mechanisms of the different scale types. We assumed the needle punctured through each scale but it is also possible that some scales cracked or fractured in response to the needle, or that the needle tilted or slipped before puncturing the scale material (Zhu et al. 2012). Future research examining the material properties and failure modes of scales in fish species that possess multiple scale types may shed additional light on this question.

Although tuberculate scales may not increase puncture resistance, they may provide other functional advantages. Tuberculate scales have many projections emanating in multiple directions, as opposed to the posteriorly-oriented projections on scales with ctenii. These projections likely increase the frictional forces that keep sediment in place when a fish is buried in the substrate (Spinner et al. 2016). It is also possible that tuberculate scales increase the ability of the integument to withstand shear forces, a force applied in a direction parallel to the material. Many predators violently shake their heads back and forth as a means to process a prey item after it is caught (Whitenack and Motta 2010; McNeil et al. 2016); during this kind of attack, the teeth of the predator would apply shear forces, rather than puncture forces, on the surface of the prey. Alternately, tuberculate scales may provide protection from larger particulate, gravel, commonly found near shore habitats, where this species can often be found (Toft et al. 2007).

## Supplementary Material

obz005_Supplementary_Data
