# Supplementary material for: Scale Thickness Predicts Skin Puncture-Force Resistance in Three Pleuronectiform Fishes
Source: Integr Org Biol. 2019 Apr 8;1(1):obz005. doi: 10.1093/iob/obz005 (PMC7671105; doi:10.1093/iob/obz005)
Supplement: obz005_Supplementary_Data [file obz005_supplementary_data.zip › Flatfish Paper Supplemental Material.docx]

**Supplemental Table 1:** **There are no differences between the eyed and blind sides of the flatfishes for any of the variables investigated.** Results of the paired samples t-tests for each species for each variable.

| Species | Variable | t | df | p-value |
| --- | --- | --- | --- | --- |
| *Parophrys* | Force | -0.545 | 9 | 0.6 |
| *Parophrys* | Area | 0.934 | 7 | 0.38 |
| *Parophrys* | Diameter | -0.644 | 7 | 0.54 |
| *Parophrys* | Thickness | 0.132 | 7 | 0.9 |
| *Isopsetta* | Force | -1.226 | 9 | 0.25 |
| *Isopsetta* | Area | 0.156 | 5 | 0.88 |
| *Isopsetta* | Diameter | 0.084 | 5 | 0.94 |
| *Isopsetta* | Thickness | -0.703 | 5 | 0.51 |
| *Platichthys* | Force | -0.307 | 11 | 0.76 |
| *Platichthys* | Area | 1.793 | 8 | 0.11 |
| *Platichthys* | Diameter | 1.049 | 8 | 0.33 |
| *Platichthys* | Thickness | 1.374 | 7 | 0.21 |

**Supplemental Figure 1:** **Descriptions of how morphometric measurements were taken on scales.** All images represent samples taken from *Isopsetta isolepis*. A shows the axis by which scale diameter (SD) was measured. B shows the shaded area for how scale area (SA) was determined. C is a histological section and represents how scale thickness (ST) was measured.
